# A222 BILIARY ATRESIA IN BRITISH COLUMBIA: THE ROLE OF REFERRAL AGE AND DIAGNOSITIC EVALUATION ON OUTCOME

**DOI:** 10.1093/jcag/gwab049.221

**Published:** 2022-02-21

**Authors:** M Smyth, R Baird, R Schreiber

**Affiliations:** Pediatric Gastroenterology, The University of British Columbia, Vancouver, BC, Canada

## Abstract

**Background:**

Biliary atresia (BA), a newborn liver disease, is the leading cause of cirrhosis and liver-related death in children and the most common indication for pediatric liver transplantation (LT). The current standard of BA care is sequential surgery with an initial Kasai hepatoportoenterostomy (KP), followed by LT for those who progress to liver failure. Survival with native liver (SNL) correlates to infant age at KP with best outcome at early KP at <30 days of age. Novel screening tests and diagnostic algorithms have been proposed, however the variability of clinical presentation and lack of a diagnostic test challenge early diagnosis and timely KP.

**Aims:**

To assess age at BA presentation and subsequent investigations to the timing of KP and outcome.

**Methods:**

A retrospective study of all BA cases referred to BC Children’s Hospital January 1, 2000-December 31, 2018. Data collection included age at referral, clinical presentation, laboratory and imaging studies, age at KP and LT. SNL and overall survival rates were determined. Descriptive statistics and data analyses using SSPS were applied.

**Results:**

In this 19-year study, there were 48 cases (58% female) of BA in BC (1:17,000 live births). KP was performed in 41 patients and 7 had primary LT. Following the initial KP, 23 cases had LT. Median age at presentation decreased from 55 to 42 days after introduction of the BC BA stool card screening program in 2014. The Median (IQR) age at KP was 62 days (48–87). Median delay from the age at first encounter to the KP was 10 days (4–21); early referral (youngest 3^rd^ of cohort) had a mean delay to KP of 25 days (15–40) compared with the late group (oldest 3^rd^ of cohort) with median delay to KP of 5 days (1–8). There were 2.4 and 1.4 investigations/patient before undergoing KP in the youngest and oldest age at presentation cohorts. HIDA scan was done in 41% and 19% of the youngest and oldest presentation cohorts respectively. Median LT age was 9.6 months (8-13months). Median age at KP for patients who received LT was 77 days (53–92), compared to 52 days (41–79) in those without LT (p=0.08). All KPs were completed by 7 surgeons, each completing 1- 10 KPs. Overall patient survival and SNL were 98% and 37.5%.

**Conclusions:**

In this cohort, SNL rates were below SNL rates in other national studies. SNL rates were higher in patients who underwent KP at a younger age, and early findings from a provincial screening program show a shift in age at presentation, potentially owing in part to increased community awareness of BA. A diagnostic algorithm that accounts for age at presentation is needed achieve timely KP.

**Funding Agencies:**

None

